# Determination of the Optimal Concentration of Valproic Acid in Patients with Epilepsy: A Population Pharmacokinetic-Pharmacodynamic Analysis

**DOI:** 10.1371/journal.pone.0141266

**Published:** 2015-10-20

**Authors:** Hiroo Nakashima, Kentaro Oniki, Miki Nishimura, Naoki Ogusu, Masatsugu Shimomasuda, Tatsumasa Ono, Kazuki Matsuda, Norio Yasui-Furukori, Kazuko Nakagawa, Takateru Ishitsu, Junji Saruwatari

**Affiliations:** 1 Division of Pharmacology and Therapeutics, Graduate School of Pharmaceutical Sciences, Kumamoto University, Kumamoto, Japan; 2 Department of Neuropsychiatry, Graduate School of Medicine, Hirosaki University, Hirosaki, Japan; 3 Center for Clinical Pharmaceutical Sciences, Kumamoto University, Kumamoto, Japan; 4 Kumamoto Saishunso National Hospital, Koshi, Japan; 5 Kumamoto Ezuko Ryoiku Iryo Center, Kumamoto, Japan; Queen's University Belfast, UNITED KINGDOM

## Abstract

Valproic acid (VPA) is one of the most widely prescribed antiepileptic drugs for the treatment of epileptic seizures. Although it is well known that the doses of VPA and its plasma concentrations are highly correlated, the plasma concentrations do not correlate well with the therapeutic effects of the VPA. In this study, we developed a population-based pharmacokinetic (PK)-pharmacodynamic (PD) model to determine the optimal concentration of VPA according to the clinical characteristics of each patient. This retrospective study included 77 VPA-treated Japanese patients with epilepsy. A nonlinear mixed-effects model best represented the relationship between the trough concentrations of VPA at steady-state and an over 50% reduction in seizure frequency. The model was fitted using a logistic regression model, in which the logit function of the probability was a linear function of the predicted trough concentration of VPA. The model showed that the age, seizure locus, the *sodium channel neuronal type I alpha subunit* rs3812718 polymorphism and co-administration of carbamazepine, clonazepam, phenytoin or topiramate were associated with an over 50% reduction in the seizure frequency. We plotted the receiver operating characteristic (ROC) curve for the logit(Pr) value of the model and the presence or absence of a more than 50% reduction in seizure frequency, and the areas under the curves with the 95% confidence interval from the ROC curve were 0.823 with 0.793–0.853. A logit(Pr) value of 0.1 was considered the optimal cut-off point (sensitivity = 71.8% and specificity = 80.4%), and we calculated the optimal trough concentration of VPA for each patient. Such parameters may be useful to determine the recommended therapeutic concentration of VPA for each patient, and the procedure may contribute to the further development of personalized pharmacological therapy for epilepsy.

## Introduction

The overall mortality rate among patients with epilepsy in high-income countries is two to five times higher than that in the general population, and the rate is further increased (up to 37 times) in low-income countries [1]. Excess mortality is highest during the first years after seizure onset, mainly related to the underlying causes of epilepsy and comorbidities [1]. Although more than 20 antiepileptic drugs (AEDs) have been used to control seizures in patients with epilepsy, one-third of patients are resistant to treatment [1, 2]. Therapeutic drug monitoring (TDM) has often been performed during the pharmacological treatment of epilepsy, but the plasma levels of some AEDs do not correlate well with the doses and/or the therapeutic or toxic effects of the drugs [2, 3]. Therefore, the pharmacological treatment of epilepsy has been empirical and often based on trial and error (e.g., increase or decrease the doses of AEDs, switch or add another AED) [2]. We considered that the establishment of a procedure to determine the optimal concentration of AEDs based on the patient’s characteristics would be useful for the pharmacological treatment of epilepsy.

Valproic acid (VPA) is one of the most widely prescribed AEDs for the treatment of both generalized and partial seizures because of its multiple mechanisms of action and acceptable safety profile [2–4]. Since the dose requirements for VPA are highly variable and interactions with other drugs are common, TDM is commonly performed during VPA therapy [2–4]. Pharmacokinetic (PK) studies of VPA revealed a wide inter-individual variability in its clearance (CL), which may be related to its dose-dependent properties, and it is considered that the development of a PK model for the estimation of VPA CL using routine clinical data contributes to dose adjustments [5–7]. Previous evidence using PK models has suggested that the daily dose of VPA, the patient age, gender, body weight, and the co-administration of carbamazepine (CBZ), phenytoin (PHT), phenobarbital (PB) and/or clobazam (CLB) affect the VPA CL [5–8]. The estimation of the individual CL for patients taking VPA by these population PK models could be used to recommend optimal individual doses based on the therapeutic range, e.g., 50–100 μg/ml, determined based on the results of a previous small study [9]. However, several reports have failed to find a high correlation between the plasma VPA concentrations and the therapeutic or toxic effects of the drug [4, 10, 11]. Thus, the determination of the optimal concentration of VPA for each patient based on the combined model of PK and pharmacodynamics (PD) would be useful for VPA therapy.

We previously demonstrated that the *sodium channel neuronal type I alpha subunit* (*SCN1A*) IVS5-91 G>A polymorphism (rs3812718) affected carbamazepine (CBZ)-resistant epilepsy in a cross-sectional study of Japanese patients with epilepsy being treated with CBZ, including those with co-administered VPA [12]. This association was replicated in two recent studies [13, 14], but not in all studies [15]. CBZ exerts its PD effects by blocking voltage-gated Na+ channels, such as Nav1.1 [2, 3]. The *SCN1A* gene encodes the alpha-subunit of Nav1.1, and the A allele has been shown to generate alternative splicing products, which results in altered proportions of neonate and adult exon 5 transcripts in brain tissue [16, 17]. One of the mechanisms of action of VPA is blocking the Na+ channel, similar to CBZ [2, 3]. Therefore, we speculated that the *SCN1A* genotype may also be associated with a reduced the therapeutic effect of VPA through the alteration of the response of the Na+ channel.

Our recent study developed an equation to describe the relationship between the serum VPA concentrations and the risk of γ-glutamyltransferase elevation by developing population PK and PK-PD models using a non-linear mixed-effect model (NONMEM) program [8]. The present study aimed to develop a population-based PK-PD model for the relationship between the VPA concentration and the reduction in seizure frequency using a similar procedure. In this study, we applied population PK-PD modeling to determine the optimal concentration of VPA according to the clinical characteristics of individual epileptic patients using a NONMEM program. The primary objective of this retrospective study was to evaluate the impact of the clinical characteristics of patients, including the *SCN1A* rs3812718 polymorphism, on the relationship between the serum VPA concentration and the control of seizures during long-term VPA therapy.

## Methods

### Subjects and study protocol

The present study was conducted among the same epileptic patients reported in our previous study [8]. All patients were treated at Kumamoto Saishunso National Hospital (Kumamoto, Japan) between June 1989 and April 2011. The patients fulfilled all of the following conditions: had been receiving sustained-release VPA for three weeks or longer and achieved steady-state plasma concentrations of VPA; were not taking any drugs that might alter the CL of VPA except for other AEDs; did not have idiopathic epilepsy; were not severely retarded; had not discontinued the administration of VPA because of side effects; and had detailed medical data. Informed consent, including a statement regarding the privacy policy, was obtained in writing from each patient and/or his/her parent before entry into the study. This retrospective study was approved by the ethics committees of Kumamoto Saishunso National Hospital and the Faculty of Life Sciences, Kumamoto University (Kumamoto, Japan).

For all patients, the most appropriate AED was chosen according to the treatment guidelines of the Japan Epilepsy Society. The treatment was changed to another drug if the seizures remained uncontrolled, if drug-precipitated seizures were suspected or if the patient had any intolerable adverse drug reaction(s). For all patients, VPA was initiated at a dose of 15–40 mg/kg/day for children (400–1,200 mg/day for adults) and was escalated at weekly intervals by 5–10 mg/kg/day (for children) or 200 mg/day (for adults) for each step up to the maximum tolerated dose.

### Definitions

At each follow-up visit, the patients’ medical information was retrospectively obtained from their medical records. For every patient, the data included demographic information, the VPA dose and schedule, diagnoses, seizure frequency and concomitant medications, which were evaluated at every visit during VPA therapy. The types of seizures and epileptic syndromes were classified according to the guidelines of the International League Against Epilepsy [18]. The seizure frequency was monitored by means of an interview performed by the medical staff at every visit; the baseline seizure frequency was derived from the average of three to five visits before the start of VPA therapy. The efficacy (i.e., PD parameter) was evaluated on the basis of whether there was an over 50% reduction in seizure frequency (irrespective of seizure severity) compared with the baseline frequency before starting VPA therapy during a period at least five times longer than the seizure interval of each patient, according to previous studies [12, 19].

### Genotyping

Genomic DNA was isolated from EDTA blood samples using a DNA extractor WB kit (Wako Pure Chemical Industries, Ltd. Osaka, Japan). Samples were genotyped for the *SCN1A* IVS5-91 G>A polymorphism (rs3812718) using a custom-designed TaqMan-based allelic discrimination assay (Applied Biosystems) [16]. To ensure the genotyping quality, we included DNA samples as internal controls, hidden samples of a known genotype, and negative controls (water). The genotype distribution was tested for Hardy-Weinberg equilibrium using the χ^2^ test. The test was performed using the R software program (version 3.0.0; R Foundation for Statistical Computing, Vienna, Austria). A *P* value < 0.05 was considered to be statistically significant.

### Evaluation of the predicted trough concentration of VPA

The predicted trough concentration of VPA for each patient with epilepsy was determined using the population PK model for the VPA concentration constructed in our previous study [8]. The PK model was developed using a total of 827 steady-state VPA concentrations and a one-compartment model with first-order absorption and lag time was used to select the structural model for VPA [8]. A regression model was developed using the forward-inclusion and backward-elimination methods [8]. The interval between the time of the last dose and the sampling time was distributed over 24 hours [8]. The population PK model for VPA was as follows:

$$\;Vd=\;110\;\times \;{\left(\frac{Dose}{1000}\right)}^{1.51}$$ (1)

$$CL\;=\;0.577\;\times \;\left(\frac{Dose}{1000}\right)0.535\;\times \;0.875female\;\times \;1.22CBZ\;\times \;1.10PB\;\times 1.40PHT\;\times \;0.915CLB$$ (2)

where “Dose” is the daily VPA dose (mg/day); female = 1, male = 0; CBZ, PB, PHT or CLB = 1 if each drug was co-administered, and 0 if it was not [8].

### PK-PD modeling

The analyses used during the population PK-PD modeling were carried out with a NONMEM software program (NONMEM, version 7.2.0; ICON Dev Soln, Ellicott City, MD), and laplacian estimation was used for the development of the PK-PD model. The individual PK parameters, determined from our previously reported population PK model [8], were fixed in the PK-PD analysis. A logistic regression model was used to relate the probability of having an over 50% reduction in the seizure frequency. The predicted trough concentration of VPA was used as the exposure variable, and the concentration of each patient was predicted from the population PK model [8]. The probability, Pr, of having an over 50% reduction in the seizure frequency was expressed as the inverse logit function:

$$\text{Pr}=\frac{e^{logit\;\left(\text{Pr}\right)}}{1+\;e^{logit\;\left(\text{Pr}\right)}}$$ (3)

in which the logit(Pr) value is a linear function of the predictive trough concentration of VPA:

$$\begin{array}{l} Logit\left(\text{Pr}\right)\;=\;Ln\left(\frac{\text{Pr}}{1-\text{Pr}}\right)\\ \;=\;Intercept\;+\;Predictive\;trough\;concentration\;of\;VPA\;\times \;Slope\;+\;\eta \end{array}$$ (4)

where “Slope” is the slope relating the predicted trough concentration of VPA to the effect and *η* is the individual random effect.

The logit(Pr) value that described a nonlinear squared relationship of the predictive trough concentration of VPA was also tested, and the equation is described below:

$$\begin{array}{l} Logit\left(\text{Pr}\right)=Ln\left(\frac{\text{Pr}}{1-\text{Pr}}\right)\\ \;=\theta 1\;+\;{\left(Predictive\;trough\;concentration\;of\;VPA\;–\;\theta 2\right)}^{2}\times \;\theta 3\;+\;\eta \end{array}$$ (5)

where *θ1*, *θ2* and *θ3* are the invariables and *η* is the individual random effect.

The influence of covariates on the logit(Pr) value was systematically tested in the same manner as in our previous PK-PD model [8]. A regression model was developed using the forward-inclusion and backward-elimination methods. First, each covariate was incorporated nonlinearly into the basic regression model. From the basic model, important covariates were identified by plotting the estimates versus the covariates. The influence of these fixed effects was evaluated using the objective function. The full model was created by incorporating all covariates, which led to a significant decrease in the objective function. The objective function of the full model was used to test the effects of removing each covariate from the full model. Changes in the objective function of at least 3.84 [χ^2^, *P* < 0.05; degree of freedom (df) = 1] and 5.99 (χ^2^, *P* < 0.05; df = 2) were considered to be significant during the forward-inclusion and backward-elimination analyses. The covariates tested for effects on the Intercept, Slope, *θ1*, *θ2* and/or *θ3* were the patient age, gender, seizure locus, seizure type, complication with intellectual disability, co-administered AEDs [CBZ, clonazepam (CZP), CLB, gabapentin (GBP), lamotrigine (LTG), PB, PHT, topiramate (TPM) and/or zonisamide (ZNS)]. The final PK-PD model was selected from the linear model (Eq 4) and the nonlinear squared model (Eq 5) by checking the data visually.

### Model evaluation and simulations of the PK-PD parameters

A stratified nonparametric bootstrap analysis was performed to investigate the precision of the parameters of the population PK-PD model implemented in the NONMEM and Wings for NONMEM programs (version 7.2.0; http://wfn.sourceforge.net/). One thousand replicated datasets were generated by random sampling with replacement, and were stratified according to the study population to ensure a representative study population distribution using the individual as the sampling unit. The population parameters of each dataset were subsequently estimated as described for the original estimation procedure. A visual predictive check regarding the proportion of patients with a more than 50% reduction in seizure frequency was also performed using 1,000 datasets that were randomly sampled from the original dataset, and was stratified by the statistically significant covariates identified in the final model. The visual predictive check is an internal validation tool that shows how well the model predicts the data on which the model was conditioned, and it compares the dependent variables derived from the original and simulated datasets [20]. The R-based software program, Xpose (version 4.4.1), was used for the graphical visualization of the results, and the PsN tool kit (version 3.5.3) was used for the post-processing of the results. Based on the final population PK-PD parameter estimates, the probability of there being a more than 50% reduction in seizure frequency were also simulated at the steady state in 1,000 individuals. The simulation processes were performed using the NONMEM program.

### Evaluation of the optimal trough concentration of VPA

Based on the final population PK-PD parameter estimates, an optimal cut-off point of the logit(Pr) value for a more than 50% reduction in seizure frequency was determined using the receiver operating characteristic (ROC) curve. The ROC curve was plotted and the areas under the curves (AUC) with the 95% confidence interval (CI) were calculated by the R software program (version 3.0.2; R Foundation for Statistical Computing, Vienna, Austria). The value maximizing the Youden index (= sensitivity + specificity-1) was used to determine the cut-off point for the logit(Pr) value. The optimal trough concentration of VPA for each patient was evaluated using the cut-off point of the logit(Pr) value and Eqs 4 or 5.

## Results

### Clinical characteristics of the patients

Seventy-seven patients with epilepsy fulfilled all of the inclusion criteria for this study. The clinical characteristics of the patients with epilepsy are presented in Table 1. The mean duration of follow-up was 5.1 ± 4.4 years. The frequencies of the *SCN1A* G/G, G/A and A/A genotypes were 11.7%, 53.2% and 35.1%, respectively, and the frequency of the A allele was 61.7%. The observed genotype frequency distribution was consistent with the Hardy-Weinberg equilibrium (*P* < 0.05, respectively).

**Table 1:** Clinical characteristics of the patients.

Number of patients		77
Female (%)		29 (37.7)
Age (years)		15.2 ± 8.2 [0.8–36.9]
Seizure locus	Generalize	10 (13.0)
	Partial	67 (87.0)
Seizure type	Symptomatic	38 (49.4)
	Cryptogenic	39 (50.6)
Intellectual disability (%)		59 (76.6)
VPA dose (mg/day)		1120.0 ± 592.5 [50–3200]
Predictive trough concentration of VPA (mg/day)		69.3 ± 19.9 [11.8–130.1]
Observation period (years)		5.1 ± 4.4 [0–14.5]
Number of measurement point		729
Co-administration	CBZ (%)	47 (61.0)
	CZP (%)	21 (27.3)
	CLB (%)	33 (42.9)
	GBP (%)	8 (10.4)
	LTG (%)	3 (3.9)
	PB (%)	31 (40.3)
	PHT (%)	22 (28.6)
	TPM (%)	10 (13.0)
	ZNS (%)	26 (33.8)
*SCN1A* genotype	G/G (%)	9 (11.7)
	G/A (%)	41(53.2)
	A/A (%)	27 (35.1)

The data are the means ± standard deviation [range] or number (%) for categorical variables.

VPA, valproic acid; CBZ, carbamazepine; CZP, clonazepam; CLB, clobazam; GBP, gabapentin; LTG, lamotrigine; PB, phenobarbital; PHT, phenytoin; TPM, topiramate; ZNS, zonisamide; SCN1A, sodium channel neuronal type I alpha subunit.

### PK-PD modeling

The best-fitted base model for the probability of a more than 50% reduction in seizure frequency was a logistic regression model, in which the logit(Pr) value was a linear function of the individual predicted trough concentration of VPA. Table 2 shows that the effects of the tested covariates on the objective function of the PK-PD parameters regarding the probability of a more than 50% reduction in seizure frequency. During the forward-inclusion analyses, the patient age, gender, seizure locus, complication with intellectual disability, co-administered CBZ, CZP, PHT or TPM and *SCN1A* genotype were found to be significant covariates influencing the Intercept and/or Slope of the logit(Pr) values (Table 2). On the other hand, during the backward-elimination analyses, the influence of the age, seizure locus, co-administered CBZ, CZP, PHT or TPM and *SCN1A* genotype were found to be significant covariates influencing the Intercept and Slope of the logit(Pr) values (Table 2). Therefore, the patient gender and complication with intellectual disability were removed from the full covariate model.

**Table 2:** The effects of the tested covariates on the objective function of the PK-PD parameters regarding the probability of a more than 50% reduction in seizure frequency.

		Intercept				Slope			
		Forward inclusion step		Backward elimination step		Forward inclusion step		Backward elimination step	
		*DOBF*	*P*	*DOBF*	*P*	*DOBF*	*P*	*DOBF*	*P*
Female		11.16	< 0.05	0.70	NS	14.62	< 0.05	1.35	NS
Age (years)		6.46	< 0.05	42.67	< 0.05	16.64	<0.05	31.48	< 0.05
Seizure locus		8.42	< 0.05	37.91	< 0.05	9.46	< 0.05	42.29	< 0.05
Seizure type		3.05	NS			0.67	NS		
Intellectual disability		11.84	< 0.05	0.01	NS	12.45	< 0.05	1.27	NS
Co-administration	CBZ	18.73	< 0.05	58.66	< 0.05	14.52	< 0.05	46.23	< 0.05
	CZP	8.51	< 0.05	14.13	< 0.05	5.95	< 0.05	9.54	< 0.05
	CLB	0.27	NS			0.48	NS		
	GBP	0.19	NS			0.08	NS		
	LTG	1.19	NS			2.12	NS		
	PB	0.38	NS			0.00	NS		
	PHT	21.28	< 0.05	44.01	< 0.05	28.02	< 0.05	38.54	< 0.05
	TPM	17.07	< 0.05	13.91	< 0.05	19.63	< 0.05	12.2	< 0.05
	ZNS	1.19	NS			0.69	NS		
SCN1A genotype ^a^		16.98	< 0.05	28.24	< 0.05	30.92	< 0.05	52.67	< 0.05

PK, pharmacokinetic; PD, pharmacodynamic; DOBF, difference of objective function; NS, not significant; VPA, valproic acid; CBZ, carbamazepine; CZP, clonazepam; CLB, clobazam; GBP, gabapentin; LTG, lamotrigine; PB, phenobarbital; PHT, phenytoin; TPM, topiramate; ZNS, zonisamide; SCN1A, sodium channel neuronal type I alpha subunit.

^a^ G/G genotype vs. G/A genotype vs. A/A genotype.

The final population PK-PD model for the probability of a more than 50% reduction in seizure frequency was as follows:

$$Logit\left(\text{Pr}\right)=6.1+\left(\frac{Age}{10}\right)\times \;1.0\;–1.8^{CBZ}-1.2^{CZP}-5.9^{SCN1A\;GA\;genotype\;}-4.9^{SCN1A\;AA\;genotype}-\left(13.3+3.6^{PHT}+1.7^{TPM}-2.4^{partial\;seizure}-10.1^{SCN1A\;GA\;genotype}-9.5^{SCN1A\;AA\;genotype}\right)\times \;Predicted\;trough\;concentration\;of\;VPA$$ (6)

where the predicted trough concentration of VPA was simulated based on the population PK analysis [8]; partial seizure = 1 if a partial seizure was present, and 0 if partial seizures were absent; CBZ, CZP, PHT or TPM = 1 if CBZ, CZP, PHT or TPM was co-administered, otherwise the term was assigned a value of 0; *SCN1A* G/A genotype or *SCN1A* A/A genotype = 1 for carriers of each genotype, and 0 for carriers of other genotypes.

The variations among individuals in the base model and the final population PK-PD model were 12.9 and 11.3, respectively, and the final model decreased the variation among individuals by 12.4% compared to the base model.

**Fig 1:**
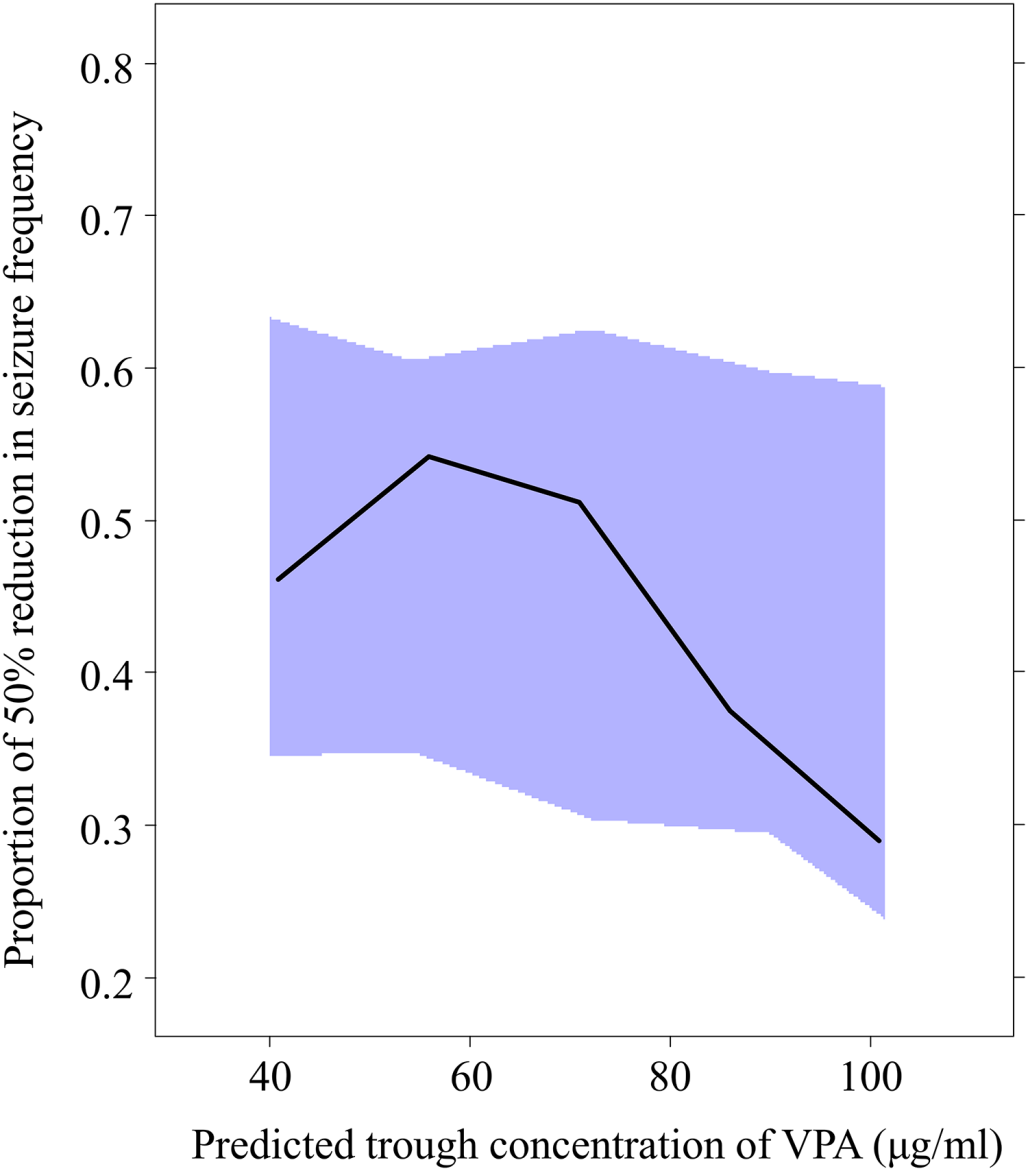
The results of the visual predictive check of the population PK-PD model. The solid line represents the observed proportion with a more than 50% reduction in seizure frequency, and the solid area represents the 95% prediction interval. PK, pharmacokinetic; PD, pharmacodynamics; VPA, valproic acid.

Among the 1,000 bootstrap runs, 972 runs exhibited successful minimization and were included in the bootstrap analysis. Table 3 shows the median parameter estimates obtained using the NONMEM program and the values with 95% CIs obtained using the bootstrap approach. The 95% CIs for all parameters obtained using the bootstrap approach were generally comparable to the estimates obtained using the NONMEM program (Table 3). A visual predictive check regarding the proportion of patients with a more than 50% reduction in seizure frequency using 1,000 datasets according to the final population PK-PD model is shown in Fig 1. The visual predictive check indicated that the final parameter estimates were reliable (Fig 1).

**Table 3:** The median PD parameter estimates of VPA in the final population PK-PD models obtained using the NONMEM program and the bootstrap analysis.

		NONMEM		Bootstrap Evaluation	
Parameter		Estimate	SE	Median	95% CI
Intercept		6.09	2.3	-5.66	0.18–13.80
	Age (years)	0.98	0.41	1.07	0.06–2.23
	CBZ	-1.75	0.50	-1.86	-3.01–-0.79
	CZP	-1.18	0.66	-1.2	-3.01–0.33
	*SCN1A* G/A genotype	-5.87	2.53	-5.51	-13.61–1.09
	*SCN1A* A/A genotype	-4.88	2.56	-4.84	-12.70–1.96
Slope		-13.5	2.3	-13.6	-31.0–-6.80
	Partial seizure	2.41	1.12	2.69	-0.53–7.82
	PHT	-3.62	1.12	-3.78	-7.10–0.36
	TPM	-1.73	1.12	-1.82	-5.42–1.90
	*SCN1A* G/A genotype	10.1	3.17	10.1	1.31–21.31
	*SCN1A* A/A genotype	9.48	3.03	9.84	2.13–20.70
Individual random effect		11.3	1.37	11.46	8.93–14.21

PD, pharmacodynamic; PK, pharmacokinetic; VPA, valproic acid; NONMEM, non-linear mixed-effect model; SE, standard error; CI, confidence interval; CBZ, carbamazepine; CZP, clonazepam; PHT, phenytoin; TPM, topiramate; SCN1A, sodium channel neuronal type I alpha subunit.

### Evaluation of the optimal trough concentration of VPA for each patient

**Fig 2:**
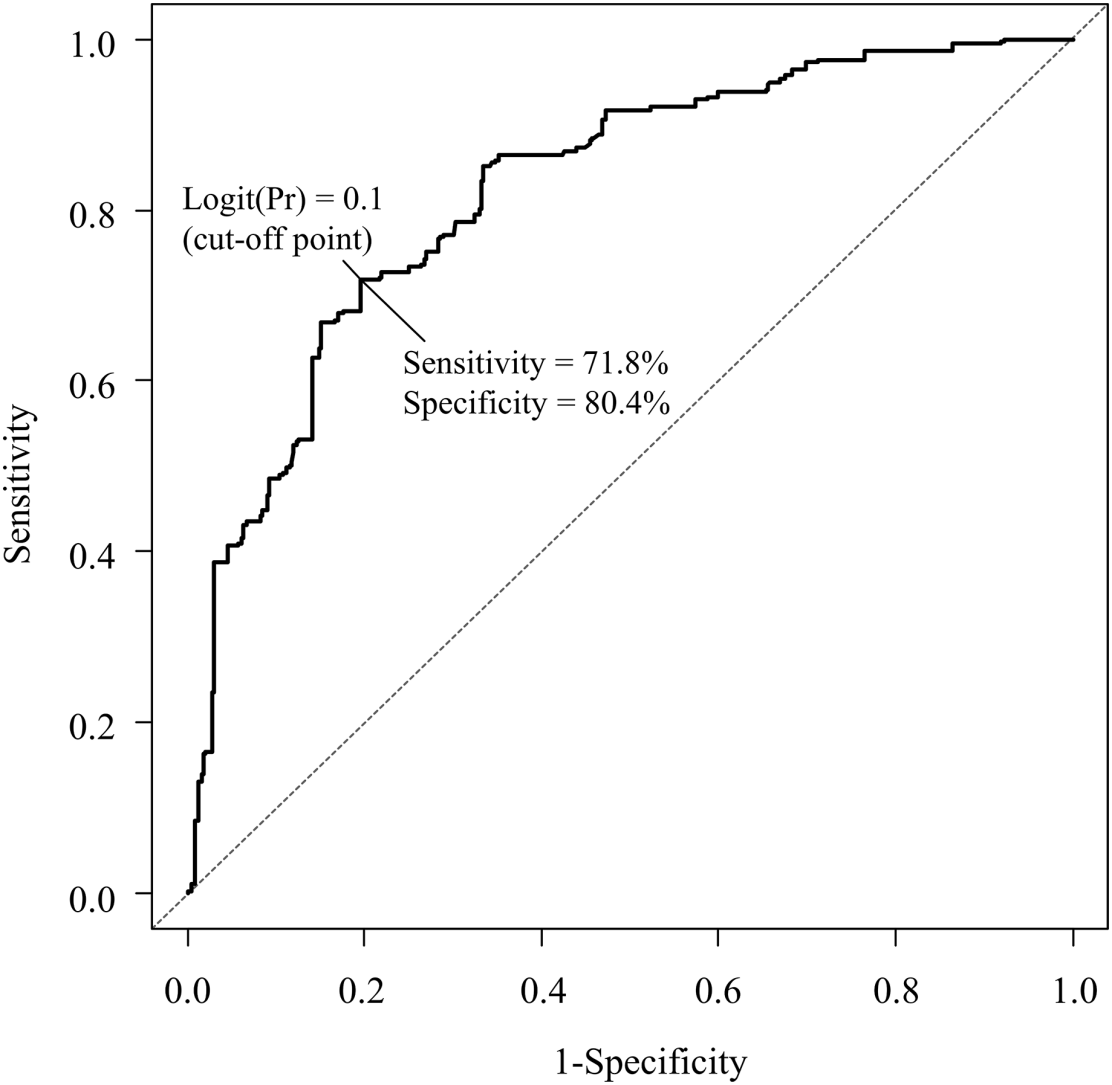
The ROC curve for the logit(Pr) value of the population PK-PD model and the presence or absence of a more than 50% reduction in seizure frequency. The solid line represents the logit(Pr) value of the final population PK-PD model. The dotted line represents the reference line. ROC, receiver operating characteristic; PK, pharmacokinetic; PD, pharmacodynamics.

Fig 2 shows the ROC curve regarding the logit(Pr) value of the final population PK-PD model (Eq 6) and the presence or absence of a more than 50% reduction in seizure frequency. The AUC of the ROC curve was 0.823 (95% CI 0.793–0.853). Using the ROC curve, a logit(Pr) value of 0.1 was selected as the optimal cut-off point, which had a sensitivity of 71.8% and a specificity of 80.4% (Fig 2). Based on the cut-off point of the logit(Pr) value, i.e. 0.1 and Eq 6, we calculated the optimal trough concentration of VPA in each patient. The specific examples of the optimal trough concentrations of VPA for simulated patients are shown in Table 4.

**Table 4:** Specific examples of the optimal trough concentration of VPA for simulated patients.

	Age	Seizure type	Co-administrated AED	*SCN1A* genotype	Optimal trough concentration of VPA
Case 1	5	Generalized	PHT	G/G	63.5 μg/ml
Case 2	5	Generalized	PHT	G/A	71.3 μg/ml
Case 3	5	Generalized	PHT	A/A	78.5 μg/ml
Case 4	10	Generalized	PHT	G/G	92.0 μg/ml
Case 5	10	Generalized	PHT	G/A	140.9 μg/ml
Case 6	10	Generalized	PHT	A/A	142.4 μg/ml

VPA, valproic acid; AED, antiepileptic drug; PHT, phenytoin; SCN1A, sodium channel neuronal type I alpha subunit.

### Effect of the patient age on the PK-PD modeling

Since we included patients of 0–36 years of age in the present study, we conducted separate PK-PD analyses of the patients who were 18 years of age or younger (n = 56; points of measurement: 597; mean age: 11.7 ± 4.41 years) and those who were 19 years of age or older (n = 21; points of measurement: 132; mean age: 26.16 ± 5.34 years). The final population PK-PD models for the probability of a more than 50% reduction in seizure frequency were as follows:

In the subjects aged 18 years or younger:

$$Logit\left(\text{Pr}\right)=7.73\;–\;4.88^{CBZ\;}–\;1.93^{PB}\;–\;4.75^{SCN1A\;GA\;genotype}\;–\;4.30^{SCN1A\;AA\;genotype}\;–\;\left(10.9\;–\;4.73^{CBZ}\;+\;3.86^{PHT}\;–\;7.62^{SCN1A\;GA\;genotype}\;–\;7.60^{SCN1A\;AA\;genotype}\right)\;\times \;Predicted\;trough\;concentration\;of\;VPA$$ (7)

In the subjects aged 19 years or older:

$$Logit\left(\text{Pr}\right)=10.3\;–2.56^{CZP}\;–\;9.88^{SCN1A\;GA\;genotype}\;–\;14.3^{SCN1A\;AA\;\text{genotype}}\times \;Predicted\;trough\;concentration\;of\;VPA$$ (8)

where the predicted trough concentration of VPA was simulated based on the population PK analysis [8]; partial seizure = 1 if a partial seizure was present, and 0 if partial seizures were absent; CBZ, PB, CZP or PHT = 1 if CBZ, PB, CZP, PHT or was co-administered, otherwise the term was assigned a value of 0; *SCN1A* G/A genotype or *SCN1A* A/A genotype = 1 for carriers of each genotype, and 0 for carriers of other genotypes. The variations among individuals of the PK-PD models of the patients aged 18 years or younger and those aged 19 years or older were 11.5 and 9.71, respectively.

## Discussion

To the best of our knowledge, this is the first report to develop an equation regarding the relationship between the serum VPA concentrations and the seizure control by developing a population PK-PD model (Eq 6). Moreover, we determined the optimal cut-off point of the logit(Pr) value for a more than 50% reduction in seizure frequency and were able to evaluate the optimal trough concentration of VPA for each patient (Fig 2 and Table 4). This information may be utilized for setting an upper limit of the trough concentration of VPA, which can control seizures for each patient with epilepsy. In addition, the procedure using the PK-PD model may contribute to the further development of personalized pharmacological therapy for epilepsy.

The pharmacological treatments of epilepsy have been empirical and often based on trial and error [2]. The population-based therapeutic range of VPA, e.g., 50–100 μg/ml, is only a rough adjunctive guide to efficacy [9]; measurements in the clinical setting are the most useful for assessing compliance [3]. Indeed, although the plasma concentrations and doses of VPA are highly correlated [9], the concentrations and biological effects are not [4, 10, 11]. Therefore, it is considered that the optimal VPA concentrations differ among individual patients with epilepsy, and the determination of the optimal serum VPA concentration to achieve seizure control is required for each patient. The present study makes it possible to evaluate the optimal trough concentration of VPA in patients with more than 50% reductions in seizure frequency for individual patients based on a population PK-PD model (Eq 6). For the patients who received VPA but had poorly controlled seizures, even if the dose of VPA were increased over the estimated value of the optimal trough concentration, a reduction in seizure frequency would not be expected. Therefore, evaluating the optimal trough concentration of VPA using a population PK-PD model may be useful for determining the therapeutic strategy (e.g., increase the dose of VPA, switch or add another AED).

In the human brain, VPA acts via several mechanisms, including the potentiation of the inhibitory activity of gamma-aminobutyrate (GABA), attenuation of N-methyl-D-aspartate-mediated excitation and blocking of Na+, Ca2+ and voltage-gated K+ channels [2, 3]. Although the mechanisms underlying drug resistant epilepsy are still not fully understood, it is considered that alterations in the structure and/or function of the drug target is associated with drug resistant epilepsy due to decreased therapeutic effects of the AEDs [2]. In the present study, the *SCN1A* G/A or A/A genotype carriers were associated with a decreased intercept of the logit(Pr) value (5.9 or 4.9, respectively) and a decrease in the slope of the logit(Pr) value (10.1 or 9.5, respectively), suggesting that the *SCN1A* A allele was associated with decreased therapeutic effects of VPA (Eq 6 and Table 2). In our previous cross-sectional study of patients with epilepsy, the *SCN1A* A/A genotype was also associated with CBZ-resistant epilepsy, including in patients co-administered VPA [12].

The *SCN1A* gene encodes the alpha subunit Nav1.1 of the voltage-gated Na+ channel and plays a crucial role in the pathogenesis of several monogenic epilepsy syndromes, including genetic epilepsy with febrile seizures plus (GEFS+) and Dravet syndrome [21, 22]. The *SCN1A* rs3812718 polymorphism is located in the splice-donor site of exon 5 of the *SCN1A* gene and is associated with the alternative splicing of *SCN1A* exon 5 [16]. Moreover, the genetic findings are supported by data showing that the A allele of patients with the *SCN1A* rs3812718 polymorphism leads to reduced expression of the neonatal exon 5N relative to the adult exon 5A [16, 17], which can alter the electrophysiological properties of Nav1.1 *in vitro* [23]. Therefore, the *SCN1A* A/A genotype may be associated with treatment-resistant epilepsy in patients administered VPA due to the different expression levels of the neonatal and adult isoforms.

In the present study, the patient age and seizure locus were associated with the probability of a more than 50% reduction in seizure frequency in the population PK-PD model (Eq 6 and Table 2), and these associations were consistent with a previous report [24]. Co-administration of CBZ, CZP, PHT, or TPM was associated with a decreased logit(Pr) value [1.8 (intercept), 1.2 (intercept), 3.6 (slope) or 1.7 (slope), respectively] in the PK-PD model (Eq 6). CBZ, PHT and TPM all block the Na+ channels, similar to VPA [2], suggesting that combining VPA with CBZ, PHT and/or TPM may not provide additional therapeutic effects. On the other hand, the mechanisms underlying the association between the co-administration of CZP and the probability of a more than 50% reduction in seizure frequency are currently unclear, and further investigations are needed.

There are limitations to the present study that should be noted. First, a major limitation of this study was its retrospective study design and the fact that it investigated a small number of patients, which may have resulted in a lack of power. In addition, we included only Japanese subjects; therefore, it is unclear whether our results can be generalized to other populations, such as Caucasian populations. Thus the current findings need to be verified in prospective study conducted among a larger patient population, which includes patients from other ethnic groups. Second, we developed the PK-PD model for the probability of seizure reduction using a controlled release VPA treatment that followed first-order kinetics. We also attempted to develop a PK model using a controlled-release VPA treatment that followed zero-order kinetics; however, the zero-order kinetic model reflected the original PK data less accurately than the first-order kinetic model. Further studies with larger numbers of patients may be needed to develop a model using a zero-order kinetic model. Nevertheless, the PK-PD results of the bootstrap analysis also showed that the median parameter estimates obtained from 972 bootstrap data sets were generally comparable with the estimates obtained using the NONMEM program (Table 3), and the visual predictive check among 1,000 datasets indicated that the final parameter estimates were reliable (Fig 2). Third, we determined the optimal trough concentration of VPA based on the results of the efficacy of VPA therapy, but did not include the results for the adverse effects of VPA. Although VPA therapy is relatively safe [2–4], the National Institutes of Health warn patients receiving VPA about the risk of severe damage to the liver and pancreas [25]. In addition, long-term treatment with VPA has been associated with hepatotoxicity, mitochondrial toxicity, hyperammonemic encephalopathy, hypersensitivity syndrome reactions, neurological toxicity, metabolic and endocrine adverse events and teratogenicity [25]. Further analyses based on both the efficacy and adverse effects should be performed to determine the recommended therapeutic concentration for each patient. Fourth, we included patients who were from 0 to 36 years of age in the present study. We also conducted separate PK-PD analyses in the patients who were 18 years of age or younger and in those who were 19 years of age or older (Eqs 7 and 8). Although the *SCN1A* genotypes were found to be significant covariates that influenced the logit(Pr) values in the both models, the extent of the impact of the *SCN1A* genotype and the co-administrated AEDs on the logit(Pr) values differed between these models (Eqs 7 and 8). Therefore, there is a possibility that the patient age may affect the PK-PD model. However, the patient population of the present study was small, especially that of the patients who were 19 years of age or older, thus further studies with larger numbers of patients are needed to verify the influence of patient age on the findings of the present study. Finally, although the study subjects were not taking any drugs that might alter the CL of VPA (with the exception of other AEDs), we could not completely exclude the possible effects of several potential environmental factors, such as coffee intake [26], on the CL of VPA.

In conclusion, this study makes it possible to evaluate the optimal concentration of VPA to achieve reductions in the seizure frequency for each patient using a population PK-PD modeling approach. The procedure may be useful to determine the recommended therapeutic concentration of AEDs for each patient, and may contribute to the further development of personalized pharmacological therapy, although a prospective study in a larger patient cohort is necessary to evaluate the predictive capacity of this model.
